# The Comparative Effectiveness of Monotherapy and Combination Therapies: Impact of Angiotensin Receptor Blockers on the Onset of Alzheimer’s Disease

**DOI:** 10.14283/jarlife.2023.8

**Published:** 2023-06-20

**Authors:** Y. Wang, M. Li, L.E. Kazis, W. Xia

**Affiliations:** 1 Geriatric Research Education and Clinical Center, Bedford VA Healthcare System, Bedford, MA, USA; 2 Department of Mathematical Sciences, Bentley University, Waltham, MA, USA; 3 Center for Healthcare Organization and Implementation Research, Bedford VA Healthcare System, Bedford, MA, USA; 4 Department of Health Law, Policy and Management, Boston University School of Public Health, Boston, MA, USA; 5 Harvard Medical School and Rehabilitation Outcomes Center (ROC), Spaulding Rehabilitation Hospital, Boston, MA, USA; 6 Department of Pharmacology, Physiology & Biophysics, Boston University School of Medicine, Boston, MA, USA; 7 Department of Biological Sciences, University of Massachusetts, Lowell, MA, USA

**Keywords:** Drug repurposing, combination therapy, angiotensin-converting enzyme inhibitor, angiotensin II receptor blocker, beta blocker, metformin, statin

## Abstract

**Background:**

The criteria for use of Alzheimer’s disease (AD) drug Leqembi recommended by the Department of Veterans Affairs (VA) include patients aged 65 years or older with mild cognitive impairment (MCI) or mild AD. Comorbidities that include hypertension, hyperlipidemia, and diabetes are common among these patients.

**Objectives:**

Our objective is to investigate the comparative effectiveness of the administration of one, two, or three medications belonging to the categories of angiotensin receptor blockers (ARBs), angiotensin-converting enzyme inhibitors (ACEIs), Beta Blockers, Statins, and Metformin, for their potential to delay the clinical onset of AD and provide a window of opportunity for therapeutic intervention.

**Design:**

Retrospective matched case-control study.

**Setting:**

Data from the Department of Veterans Affairs national corporate data warehouse.

**Participants:**

We conducted an analysis of 122,351 participants (13,611 with AD and 108,740 without AD), aged 65-89, who began at least one of the prescribed medication classes under investigation between October 1998 and April 2018.

**Measurements:**

We utilized Cox proportional hazard regressions, both with and without propensity score weighting, to estimate hazard ratios (HR) associated with the use of different medication combinations for the pre-symptomatic survival time of AD onset. Additionally, we employed a supervised machine learning algorithm (random forest) to assess the relative importance of various therapies in predicting the occurrence of AD.

**Result:**

Adding Metformin to the combination of ACEI+Beta Blocker (HR = 0.56, 95% CI (0.41, 0.77)) reduced the risk of AD onset compared to ACEI monotherapy alone (HR = 0.91, (0.85, 0.98)), Beta Blocker monotherapy (HR = 0.86, 95% CI (0.80, 0.92)), or combined ACEI+Beta Blocker (HR=0.85, 95%CI (0.77, 0.94)), when statin prescribers were used as a reference. Prescriptions of ARB alone or the combination of ARB with Beta Blocker showed an association with a lower risk of AD onset.

**Conclusion:**

Selected medications for the treatment of multiple chronic conditions among elderly individuals with hypertension, hyperlipidemia, and diabetes as monotherapy or combination therapies lengthen the pre-symptomatic period before the onset of AD.

## Introduction

**T**he recent approval of the Alzheimer’s disease (AD) drug Leqembi by the Food and Drug Administration (FDA) provides a new therapy for the treatment of AD (1). Compared to the general guideline for the appropriate use of Leqembi (2), the inclusion criteria for use of this drug recommended by the Department of Veterans Affairs (VA) include patients aged 65 years or older with mild cognitive impairment (MCI) or mild AD. Comorbidities that include hypertension, hyperlipidemia, and diabetes are common among these patients, who are often prescribed one, two, or three medications belonging to the categories of angiotensin receptor blockers (ARBs), angiotensin-converting enzyme inhibitors (ACEIs), Beta Blockers, Statins, and Metformin. It is highly significant to explore potentials of these medications to delay the clinical onset of AD and provide a window of opportunity for therapeutic intervention, regardless of the eligibility of these patients for Leqembi. These drugs are added to various medications postulated as candidate treatments for AD (3, 4). Currently, more than 30 FDA-approved drugs for other indications are selected in preclinical or clinical investigations for use as AD therapies (5).

Patients diagnosed with AD often exhibit a wide range of comorbid diseases such as diabetes, hypertension, or hypercholesterolemia (6). Many of these comorbid conditions related to AD have been well-recognized as modifiable risk factors for the onset of AD (7). The renin-angiotensin system (RAS)-acting antihypertensive ARB and ACEI, Beta Blockers, Metformin, and Statin are all approved by the FDA and therefore commonly used concomitantly by patients with comorbidities. A growing body of observational studies shows that selected conventional medications for the treatment and prevention of common AD comorbidities may play a useful role in prolonging the pre-symptomatic stage of the occurrence of AD. The solo use of ARB (8, 9, 10), ACEI (11), Statins (12, 13), Beta Blockers (14), and anti-diabetic Metformin (15) were extensively explored for their association with the reduction of the risk of AD. Although the concomitant use of medications for controlling multiple comorbidities is highly prevalent among patients with AD, limited research has been conducted to examine the associations between concomitant use of these medications and the clinical onset of AD.

Previous studies regarding the concomitant use of FDA-approved drugs on the occurrence of AD found that the concomitant use of Statin and RAS-acting antihypertensive drugs, in particular ARBs, was associated with a reduced risk of AD and related dementia than concomitant use of Statins and non-RAS-acting antihypertensives (16). Our earlier study reported that prescription of Statins and ACEI together significantly reduced the risk of developing AD in patients with a history of traumatic brain injury (TBI) (17). However, none of them investigated the concomitant use of three medications using a corporate data warehouse composed of electronic medical records from 25 million subjects within the VA Healthcare System (17, 18, 19).

Due to many of those comorbid conditions being highly correlated with one another, the concurrent use of medications for those comorbid conditions is common among the elderly (20). For example, clinical guidelines suggest the utilization of concomitant medications as an initial approach to attain improved management of blood pressure (20). The class of antihypertensive agents known as ARBs is extensively employed to manage hypertension, whereas statins are employed to manage hypercholesterolemia. Approximately 25% of adults age 65 and above use antihypertensive agents and Statins concomitantly (16). The concomitant use of ARB and Statin drugs has been postulated to have the potential for therapeutic applications in those with metabolic syndrome, type 2 diabetes, stroke, and heart failure (21). Concomitant use of multiple medications with distinct underlying mechanisms has the potential to enhance the efficacy of drug repositioning through synergistic effects, as well as delay or reduction in the development of drug resistance (22). For this reason, it is imperative to conduct a comprehensive evaluation of the concurrent use of multiple medications to examine the extent of their favorable impact on the first occurrence of AD.

The high prevalence of concomitant use of medications among patients with AD provides a unique opportunity for exploring the associations of medications with the delayed onset of AD.

This study advances our understanding of the relative effectiveness of various medications and their combinations, with a particular focus on the concomitant use of medications involving up to three different classes, an area that has not been previously explored. The objective of this study is to utilize claims data obtained from a large population-based cohort to assess and compare the effectiveness of different combination therapies consisting of one, two, or three FDA-approved drugs commonly prescribed to the elderly population (ARB, ACEI, Beta Blocker, Metformin, and Statins) in delaying the onset of Alzheimer’s disease (AD).

## Methods

This study was approved by the Bedford VA Hospital Institutional Review Board. We used administrative data from the VA Informatics and Computing Infrastructure (VINCI) Resource Center derived from both inpatient and outpatient visits and patients’ prescriptions.

### Study design

This is a retrospective matched case-control study with survival endpoints. The aim of this study is to evaluate the comparative effectiveness of mono and combined therapies for delaying the occurrence of the clinical onset of AD. In the doubly robust propensity score weighted cox model, we compared the hazard risk of clinical onset of AD in individuals with the prescription of mono-or combined therapies of ACEI, ARB, Beta Blocker, Metformin, or Statins versus those without prescription of the above therapies. In the random forest model, we applied the permutation importance approach to evaluate the relative importance of these therapies in predicting the clinical onset of AD.

### Study population

Patient health records were obtained from the Department of Veterans Affairs (VA) national corporate data warehouse (VA-CDW) database. This included inpatient and outpatient visits, vital status, and patients’ prescriptions during the study period from October 1, 1998, to April 1, 2018. We only include patients whose ages were >= 65 and < 90 years old at the end of the study period. Patients with AD were identified using ICD-9 and -10 diagnosis codes (23), which are standardized medical codes for the classification of diseases and medical conditions. Subjects who received at least two AD outpatient visits or one inpatient diagnosis (ICD-9 (331.0) and ICD-10 (G30.x) codes) were defined as AD patients with the first date of an AD diagnosis as the date of AD onset. The control or comparator group was a random sampling of 10% of the subjects with records in VINCI without a claims diagnosis of AD during the study period.

### Medication prescription and adherence

**Figure 1.**
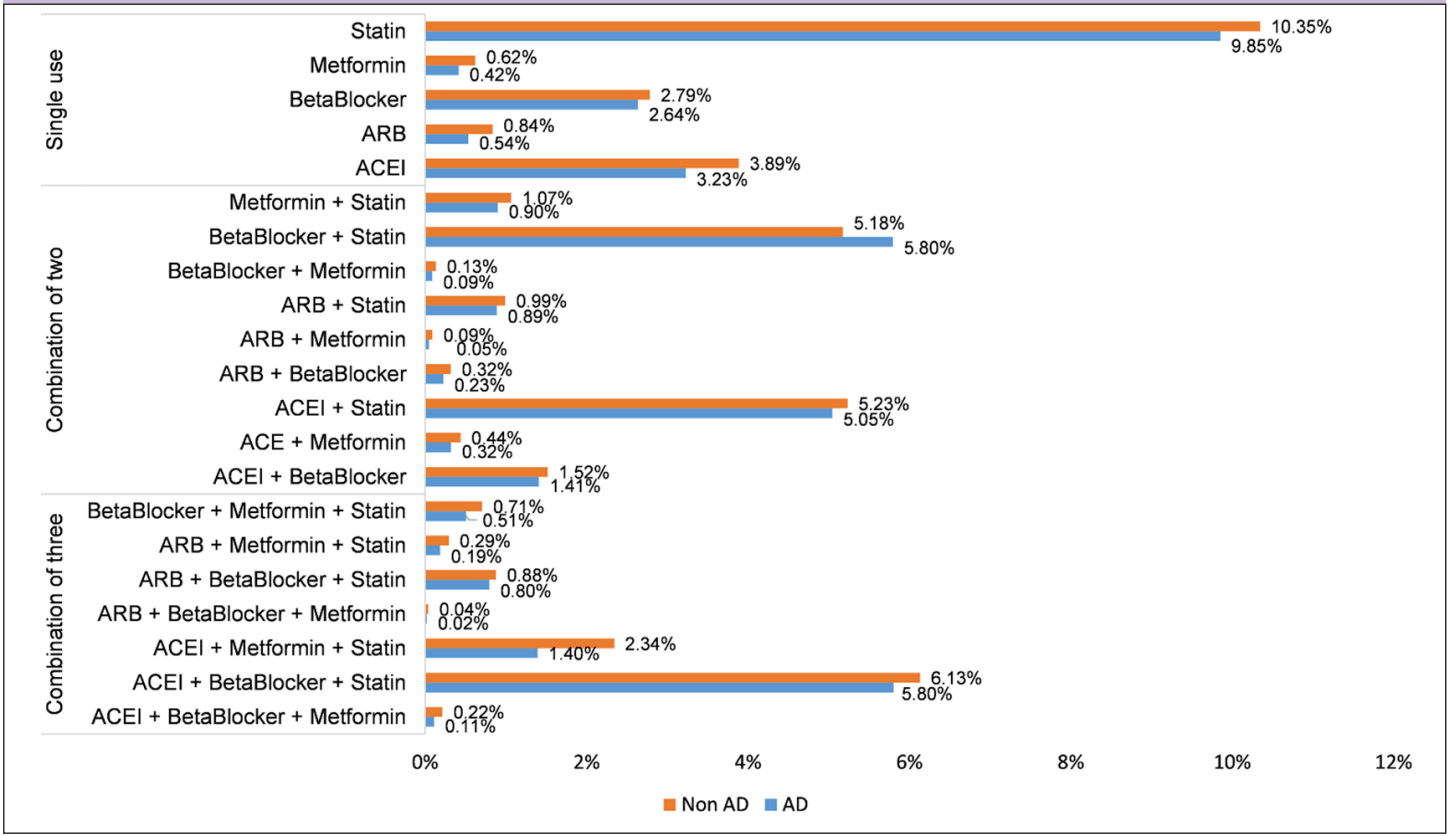
Medication usage among AD patients and control subjects

We collected patient’s medication prescription data for ACEI, ARB, Beta Blocker, Statin, and Metformin from the same database (Fig 1). “Users of medication(s)” in this paper were defined as the patients with the following two criteria: 1) those initiated the use of any of the target medications before the first diagnosis of AD, or before the censoring date in the case of the control group; 2) those who adhered to the treatment with the class-level medication possession ratio (MPR) of more than 0.80. Among qualified medication users, we analyzed single medication users and users with concomitant use of two or three medications. “Single medication users” were defined as those who used only one of the target medication classes during the study period. The “Users with concomitant use of two or three medications” were those who concomitantly prescribed the combined therapy for more than 90 days during the study period.

The graph reflects the distribution of the target medications for AD vs. non-AD group and the percentages for each medication group.

The prescription of both ACEI and ARB is not recommended in clinical practice as this combination has been postulated to have a high risk of adverse renal outcomes (24). Consistent with the recommendation, a limited number of patients (68 non-AD patients and 2 AD patients) who had a prescription of both ACEI and ARB during the study period of 20 years was identified. These patients were excluded from this study.

The average number of patients prescribed at least two classes of medications from the class of ARB, ACEI, Beta Blocker, Metformin, and Statin was 170,957 per year within the VA healthcare system. On average, more than 17% of patients have been prescribed at least two classes of medications each year (Fig 1).

### Demographics and covariates

Patients’ demographic characteristics (age, sex, race/ ethnicity), and comorbidities related to mental health and other health conditions were included as covariates to adjust for possible confounding effects (25, 26). The information on comorbidities was extracted from inpatient and outpatient visits with one ICD9/10 code and from prescription records between October 1, 1998, and April 1, 2018.

Mental health comorbidities included anxiety, bipolar disorder, schizophrenia, posttraumatic stress disorder (PTSD), depression, and substance use disorders (Table 1). Medical comorbidities included diabetes, sleep disorder, thyroid disorder, cardiac dysrhythmia, cancer, chronic heart failure, coronary artery disease, hyperlipidemia, hypertension, liver disease, lung disease, and renal failure (Table 1).

**Table 1.** Baseline characteristics of study subjects

		non-AD (108,740)	AD (13,611)
		Mean(SD)	Mean(SD)
Age		75.80 (6.82)	82.72 (5.08)
		Count (%)	Count (%)
Sex			
	Male	106,482 (97.92%)	13,365 (98.19%)
	Female	2,258 (2.08%)	246 (1.81%)
Race	White	96,921 (89.13%)	12,226 (89.82%)
	American Indian or Alaska	729 (0.67%)	67 (0.49%)
	Asian	555 (0.51%)	43 (0.32%)
	Black or African American	8,995 (8.27%)	1,080 (7.93%)
	Native Hawaiian or Other Native	977 (0.9%)	120 (0.88%)
	Unknown	563 (0.52%)	75 (0.55%)
Ethnicity	Hispanic or Latino	3,884 (3.57%)	754 (5.54%)
	Not Hispanic or Latino	102,904 (94.63%)	12,596 (92.54%)
	Unknown	1,952 (1.8%)	261 (1.92%)
Hypertension		47,289 (43.49%)	2,490 (18.29%)
Hyperlipidemia		47,148 (43.36%)	2,474 (18.18%)
Depression		18,414 (16.93%)	999 (7.34%)
Tobacco Use		18,526 (17.04%)	582 (4.28%)
Obesity		20,035 (18.42%)	728 (5.35%)
Lung disease		16,754 (15.41%)	774 (5.69%)
Coronary artery disease		16,607 (15.27%)	1099 (8.07%)
Diabetes		15,099 (13.89%)	741 (5.44%)
Anxiety		10,863 (9.99%)	519 (3.81%)
Post-traumatic stress disorder		10,957 (10.08%)	441 (3.24%)
Alcohol		9,534 (8.77%)	305 (2.24%)
Cardiac dysrhythmia		8,370 (7.7%)	392 (2.88%)
Cancer		7,679 (7.06%)	286 (2.1%)
Peripheral arterial disease		7,050 (6.48%)	326 (2.4%)
Sleep disorder		5,963 (5.48%)	211 (1.55%)
Hypothyroidism		5,294 (4.87%)	254 (1.87%)
Congestive heart failure		4,330 (3.98%)	191 (1.4%)
Renal failure		4,003 (3.68%)	130 (0.96%)
Substance use disorders		3,176 (2.92%)	96 (0.71%)
Stroke		3,668 (3.37%)	200 (1.47%)
Bipolar		3,357 (3.09%)	185 (1.36%)
Liver disease		2,032 (1.87%)	42 (0.31%)
Schizophrenia		1,764 (1.62%)	122 (0.9%)
Dementia		1,129 (1.04%)	165 (1.21%)
Traumatic brain injury		1,084 (1%)	58 (0.43%)

### Statistical methodology

Cox proportional hazard models with and without a doubly robust method with propensity score weighting (PSW) were used to assess the association of the different medication combinations with patients’ pre-symptomatic survival time to the occurrence of AD (27, 28). In our study, the pre-symptomatic survival time of AD onset was defined as the time from 65 years to the first AD diagnosis. The PSW approach was introduced to adjust for the potential confounding of baseline demographic characteristics among different medication user groups.

Our primary independent variable for evaluation is each different medication user groups. Both demographics and comorbidities were included in all models (Table 1) as covariates. Statistical significance was set at a level of 0.05, and maximum likelihood estimation was used to obtain the hazard ratio (HR) with 95% confidence intervals (CI). When comparing two groups, if the two HRs have non-overlapping confidence intervals, they are significantly different at the level of 0.05 (29). We used Statins as the reference group as it was the most prescribed medication in our study cohort.

To understand whether significant heterogeneity exists within each medication class, we performed a further analysis to compare individual drug’s with the hazard risk of clinical onset of AD. The individual medications included in this study were Losartan (ARB), Valsartan (ARB), Lisinopril (ACEI), Atenolol (Beta Blocker), Carvedilol (Beta Blocker), Metoprolol (Beta Blocker), Labetalol (Beta Blocker), Propranolol (Beta Blocker), Metformin, Atorvastatin (Statin), Pravastatin (Statin), and Rosuvastatin (Statin). The analysis was conducted among patients who took only one of the above individual medications during the study period using the Cox proportional hazard models with and without PSW. Simvastatin was used as the reference group since it was the most prescribed medication in our study population and comparable with a number of comorbidities and clinical characteristics (Fig 1).

We also applied a permutation importance measure via a random forest approach to evaluate the relative importance of all investigated therapies in predicting the clinical onset of AD and identifying which predictors have a stronger association with the response (30, 31, 32). Demographic characteristics (age, race, sex), comorbidities, and prescription information for mono-and combined therapies of interests were included as exposures in the random forest model. We chose the permutation importance over the Gini importance in this study, as the permutation importance is considered more robust and less biased when dealing with categorical variables (33). The area under the receiver operating characteristics curve (AUC) was reported to assess the accuracy of the algorithm.

## Results

### Demographic, clinical characteristics, and the medication use of the patients

Our study included 13,611 AD patients and 108,740 non-AD subjects in the VA VINCI database. The average age for non-AD patients (75.8 ± 6.8 yrs) was slightly lower than AD patients (82.7 ± 5.1 yrs) (Table 1). The majority (AD group 98.19%, non-AD group 97.92%) of the study population is male (Table 1). AD patients appear to have a higher proportion of Hispanics (AD group 5.54%, non-AD group 3.57%) (Table 1). The prevalence of the diseases among the study population at baseline, i.e. 65 years old, varies, and most subjects have hypertension, hyperlipidemia, and/or diabetes (Table 1). Overall, the proportions of different medications indicated were fairly comparable between the AD and non-AD groups, with the highest from the group using Statins alone (AD group 9.85% vs non-AD group 10.35%), followed by the group using ACEI+ Beta Blocker +Statin (AD group 5.80% vs non-AD group 6.13%), and the group using Beta Blocker +Statin (AD group 5.80% vs non-AD group 5.19%) (Fig 1).

### The use of ARB was associated with the lowest risk of AD onset among single medication users

**Figure 2.**
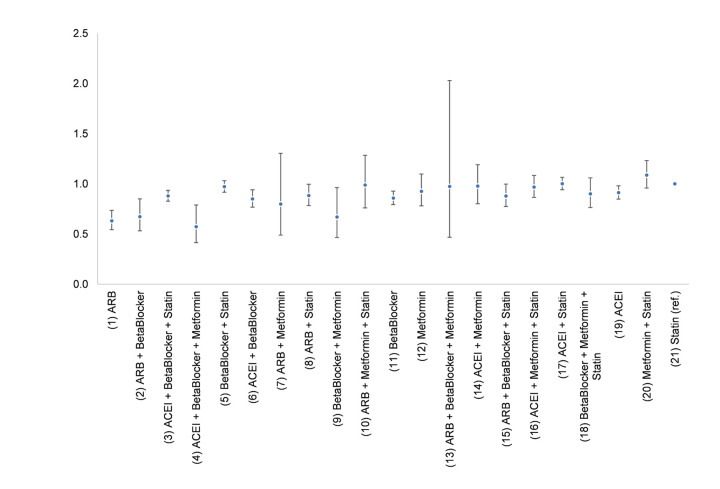
Hazard ratios of the development of AD among combined medication users with importance ranking

For patients prescribed a single medication class, the use of ARB (HR = 0.64, (0.55, 0.74)), Beta Blocker (HR = 0.86, (0.80, 0.92)), or ACEI (HR = 0.91, (0.85, 0.98)) was significantly associated with a reduced risk of AD onset compared to those prescribed with the Statin after adjustments for patients’ demographic and comorbidity characteristics (Table 2A). Patients prescribed ARBs demonstrated the strongest comparative beneficial effect with the lowest HR with PSW and highest ranking of importance with random forest (Table 2A, Fig 2). Patients prescribed Metformin (p-value = 0.164) did not reveal a statistically significant difference in the risk of AD onset compared with those who were prescribed Statins.

**Table 2.** Association of mono- or combination therapies with the development of AD (Statin as the reference)

**A. Combination**							
**Importance Ranking (Random Forest)**	**Therapy**	**Propensity Score Weighted**			**Cox**		
		**HR**	**95% CI**	**P-value**	**HR**	**95% CI**	**P-value**
1	ARB	0.63	(0.54,0.74)	< 0.001	0.64	(0.55,0.74)	< 0.001
2	ARB + Beta Blocker	0.67	(0.53,0.85)	0.001	0.67	(0.54,0.84)	0.001
3	ACEI + Beta Blocker + Statin	0.88	(0.83,0.94)	< 0.001	0.89	(0.84,0.94)	< 0.001
4	ACEI + Beta Blocker + Metformin	0.57	(0.42,0.79)	0.001	0.56	(0.41,0.77)	< 0.001
5	Beta Blocker + Statin	0.97	(0.92,1.03)	0.354	0.97	(0.92,1.03)	0.328
6	ACEI + Beta Blocker	0.85	(0.77,0.94)	0.002	0.85	(0.77,0.94)	0.001
7	ARB + Metformin	0.80	(0.49,1.30)	0.369	0.76	(0.47,1.23)	0.269
8	ARB + Statin	0.88	(0.78,1.00)	0.042	0.88	(0.78,0.99)	0.029
9	Beta Blocker + Metformin	0.67	(0.47,0.96)	0.030	0.70	(0.49,1.00)	0.051
10	ARB + Metformin + Statin	0.99	(0.76,1.28)	0.931	1.01	(0.79,1.29)	0.949
11	Beta Blocker	0.86	(0.79,0.93)	0.000	0.86	(0.80,0.92)	< 0.001
12	Metformin	0.93	(0.78,1.10)	0.375	0.89	(0.75,1.05)	0.164
13	ARB + Beta Blocker + Metformin	0.97	(0.47,2.03)	0.945	0.92	(0.46,1.85)	0.824
14	ACEI + Metformin	0.98	(0.80,1.19)	0.820	0.95	(0.78,1.15)	0.569
15	ARB + Beta Blocker + Statin	0.88	(0.77,1.00)	0.047	0.87	(0.77,0.99)	0.029
16	ACEI + Metformin + Statin	0.97	(0.86,1.08)	0.576	0.94	(0.85,1.04)	0.216
17	ACEI + Statin	1.00	(0.94,1.06)	0.963	1.00	(0.95,1.07)	0.875
18	Beta Blocker + Metformin + Statin	0.90	(0.76,1.06)	0.208	0.91	(0.78,1.06)	0.229
19	ACEI	0.91	(0.85,0.98)	0.013	0.91	(0.85,0.98)	0.008
20	Metformin + Statin	1.09	(0.96,1.23)	0.196	1.11	(0.98,1.24)	0.097
21	Statin (ref.)						
**B. Individual medication**							
		**Propensity Score Weighted Cox**			**Cox**		
		**HR**	**95% CI**	**P-value**	**HR**	**95% CI**	**P-value**
Valsartan		0.53	(0.38,0.76)	< 0.001	0.55	(0.39,0.78)	0.001
Carvedilol		0.61	(0.47,0.79)	< 0.001	0.61	(0.47,0.78)	< 0.001
Losartan		0.61	(0.51,0.73)	< 0.001	0.61	(0.51,0.72)	< 0.001
Rosuvastatin		0.65	(0.49,0.87)	0.003	0.64	(0.49,0.84)	0.001
Atorvastatin		0.70	(0.61,0.80)	< 0.001	0.64	(0.49,0.84)	0.001
Metoprolol		0.78	(0.71,0.87)	< 0.001	0.64	(0.49,0.84)	0.001
Labetalol		0.80	(0.18,3.58)	0.766	0.45	(0.11,1.87)	0.271
Lisinopril		0.87	(0.80,0.94)	< 0.001	0.85	(0.79,0.91)	< 0.001
Pravastatin		0.88	(0.77,1.00)	0.048	0.88	(0.78,1.00)	0.059
Metformin		0.88	(0.74,1.04)	0.140	0.84	(0.71,0.99)	0.040
Atenolol		0.90	(0.78,1.03)	0.120	0.87	(0.76,1.00)	0.044
Propranolol		1.17	(0.91,1.52)	0.225	1.15	(0.90,1.48)	0.269

Statin was set as the reference group for the comparison of combined medications. The importance of combined therapies in predicting AD onset was measured by the mean decrease in accuracy (from the largest to the smallest). A higher mean decrease in accuracy is indicative of a more important variable, and the combinations are ordered from top to bottom as most to least important in predicting the development of AD.

Compared to the singular use of ARB, the concomitant use of ARB with other medications did not further lower the risk of AD onset. The concomitant use of ARB + Beta Blocker (HR = 0.67, (0.54, 0.84)) and the singular use of ARB (HR = 0.64, (0.55, 0.74)) had comparable effectiveness, and both are with significantly reduced risk compared with the reference group (Statin). The concomitant use of ARB + Statin (HR = 0.88, (0.78, 0.99)) and ARB + Metformin + Statin (HR = 1.01, (0.79, 1.29)) did not appear to have a significantly more positive effect on AD onset than the singular use of ARB (Table 2A, Fig 2). The combination of ARB with Metformin had no significant difference from the reference group.

### Metformin enhanced the effect of the ACEI + Beta Blocker

We found that the concomitant use of ACEI + Beta Blocker + Metformin (ABM) was associated with a lower risk of AD onset than ACEI, Betablocker or ACEI + Beta Blocker (p=0.0495) alone. This combined therapy ABM (HR = 0.56, 95%CI (0.41, 0.77)) appeared to have lower risk than the singular use of ACEI (HR = 0.91, (0.85, 0.98)), Beta Blocker (HR = 0.86, 95%CI(0.80, 0.92)), and ACEI + Beta Blocker (HR=0.85, 95%CI (0.77, 0.94)) (Table 2A, Fig 2).

**Figure S1.**
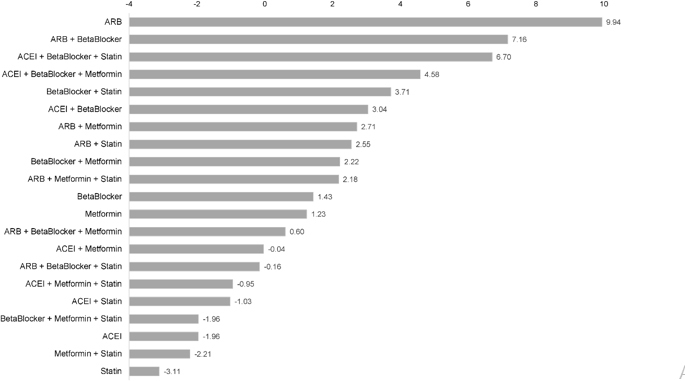
Variable importance ranking of medication combinations from the Random Forest model. The importance was measured by the mean decrease in accuracy (from the largest to the smallest). A higher mean decrease in accuracy is indicative of a more important variable, and the combinations are ordered from top to bottom as most to least important in predicting the development of AD.

**Figure S2:**
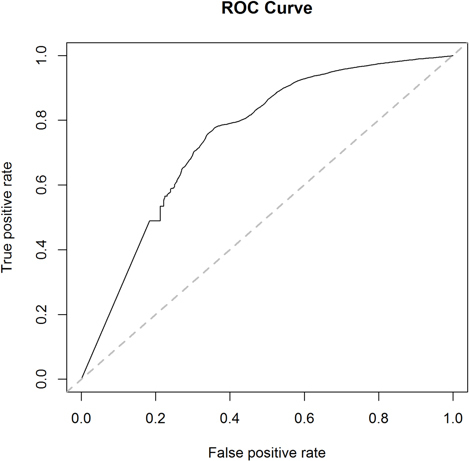
Receiver operating characteristic (ROC) curves of the random forest model that predicts the development of AD.

The result from the random forest analysis was consistent with the outcomes generated by the Cox regression analysis, as the estimated mean of the HR generally increases as the importance ranking increases (Fig 2). In addition, the reported therapies that showed significant benefits in lowering the risk of AD onset had higher importance ranking (from the random forest model) than their comparison therapies. There is no important difference between random forest and Cox regression analyses (Table 2A, Fig 2, and Fig S1). The area under the receiver operating characteristics (ROC) curve (AUC) from the random forest model (Fig S2) was 0.75, highlighting the current model properly predicting the occurrence of AD (34).

### Heterogeneity in efficacy among different medications from the same drug class on the risk of AD onset

**Figure 3.**
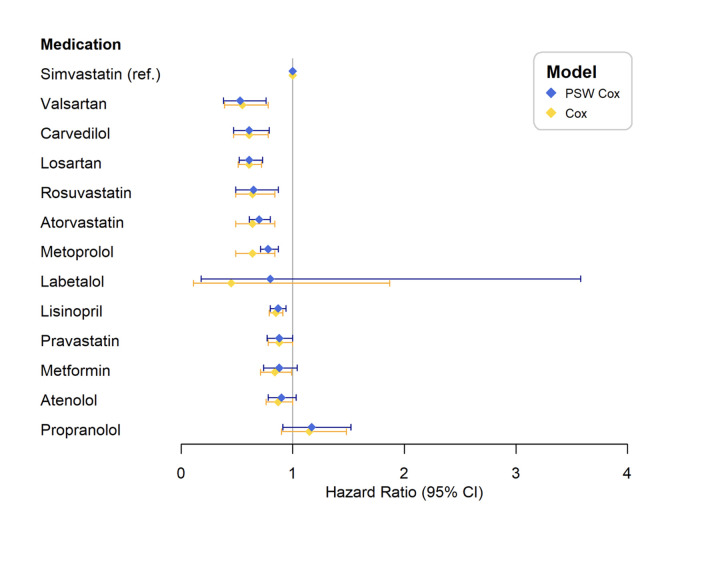
Hazard ratios (HR) of the development of AD among singular users at individual medication level

A comparison of HR of AD onset among patients prescribed single medications revealed that there was heterogeneity among different drugs within the same medication classes (Table 2B). No significant difference at the 95% CI of HRs was observed within the class of ARB (between Losartan and Valsartan) (Table 2B, Fig 3). Both ARBs (Valsartan: HR = 0.55, (0.39, 0.78) and (Losartan: HR = 0.61, (0.51, 0.72)) outperformed the use of ACEI (Lisinopril: HR = 0.85, (0.79, 0.91)), using Simvastatin users as a reference (Table 2B, Fig 3).

Simvastatin was used as a reference drug (hazard ratio = 1). All other medications discussed in this study illustrate variable hazard ratios predicting the onset of AD.

However, differences among medications in the same class were observed for Statins and Beta Blockers. Compared to the use of Simvastatin, the use of Rosuvastatin (HR = 0.64, (0.49, 0.84)), Atorvastatin (HR = 0.64, (0.49, 0.84)) or Pravastatin (HR = 0.88, (0.78, 1.00)) demonstrated significantly reduced risks of AD onset (Table 2B, Fig 3). As for Beta Blocker, Carvedilol (HR = 0.61, (0.47, 0.78)) and Metoprolol (HR = 0.64, (0.49, 0.84)) displayed lower HRs compared to Propranolol (HR = 1.15, (0.90, 1.48)) (Table 2B, Fig 3).

Patients prescribed Metformin did not reveal a significant difference in the risk of AD onset when compared with any medication under the class of Statin (Table 2B), which was consistent with the result from class-level analysis (Table 2A).

## Discussion

Aging is the primary risk factor contributing to dementia in the elderly, and successful aging, free from dementia, can be defined as a prolongation of the “pre-symptomatic” period of neurodegenerative diseases like AD with a proper cognitive reserve and brain maintenance (35). The concomitant use of multiple medications was very common for elderly patients with one or multiple comorbidities. Over 70% of patients with hypertension require at least two antihypertensive agents when blood pressure is not adequately controlled by monotherapy, particularly if subjects have comorbid conditions (36, 37). Concomitant use of multiple medications is common for many hypertension patients with comorbid conditions.

This study uses the dataset from the largest single healthcare system from the Veterans Health Administration to understand the association of mixed use of commonly prescribed medications with the risk of AD onset. We explored the comparative effectiveness of concomitant use of multiple therapies for prolongation of the pre-symptomatic survival time to AD onset, with a general tactic of repurposing existing FDA-approved drugs for other indications as an alternative approach for AD treatment. The risks (hazard ratios) of AD onset were compared among patients with one, two, or three FDA-approved medications which are widely prescribed in AD patients. Among the investigated medication classes (i.e., ARBs, ACEI, Beta Blocker, Metformin, and Statins), the greatest protective effect was associated with ARB use, followed by Beta Blocker, and ACEI, among patients who took only one of the investigated medication classes. Metformin class seemed to perform slightly better than the class of Statin medications, while the difference was not significant. There have been no other published works that have examined the use of three combination drug classes in large data bases focusing on their associations with AD onset.

ARB appeared to be the most effective monotherapy with a lower risk for onset of AD among the five investigated medication classes. A recent meta-analysis exhibited that ARB use was associated with a reduced risk of incident AD both in a randomized controlled trial and also in observational studies (10). Analysis of post-mortem brain tissue from AD or non-AD subjects suggests that ARB use was associated with fewer amyloid plaques compared to treatments with other classes of antihypertensive medications or with no medications (38). Our results corroborate our previous hypothesis of the beneficial effect of ARB use on patients with dementia (8, 39).

When comparing two first-line therapies for the treatment of hypertension, patients prescribed medications in the ARB class were associated with a lower risk of AD onset compared with those patients who were prescribed ACEI. Patients prescribed ARB medications also had a lower risk of AD onset than patients prescribed with other anti-hypertensive agents, Beta Blockers. Despite being markedly superior to Simvastatin, the combined therapy of ARBs + Beta Blockers did not show a significant additive effect on reducing the risk of AD onset when compared to the monotherapy of either ARBs or Beta Blockers.

In addition, we found that adding Metformin to the combination of ACEI + Beta blocker improved the effectiveness of the medication in lowering the risk of AD, compared with monotherapies ACEI or Beta blocker or combination of ACEI + Beta blocker. ACEI (Lisinopril), the first-line anti-hypertensive agent, and Metformin, a favored oral management of diabetes, are frequently prescribed together. Except for the major indications, these two medications have renoprotective and cardioprotective properties, respectively, and the concomitant use of Lisinopril and Metformin increased the blood glucose lowering effects of Metformin (40), while significantly decreasing blood pressure variability and mean blood pressure (41). Evidence supports the benefits of the combined therapy of ACEI + Beta Blocker in patients with a broad spectrum of cardiovascular diseases (42). As such, it is reasonable to postulate that the combination therapy of ACEI + Beta Blocker + Metformin may achieve improved management of comorbid conditions and provide additional benefits.

Our result from this study echoes the concern of reversible memory impairment among patients with Statin therapy, which makes it unadvisable to use Statins for AD patients (43, 44, 45). The FDA’s post-marketing safety surveillance system revealed several case reports of Statin-associated memory loss and improvement after discontinuation of the Statin, and issued a warning regarding the potential adverse effects of statins on cognition in 2012. Multiple reviews and meta-analyses did not indicate substantial evidence to support that Statins cause cognitive impairment to a significant degree and fail to establish the causal relationship (46, 47, 48, 49). The current understanding of Statins’ effect on cognitive function is limited by a lack of mechanism-based studies. We found that there was some heterogeneity existing within a given medication class. The HR of AD onset varies widely among Statins and Beta Blockers. Our results suggest Simvastatin appeared to be the least protective medication among Statins. Patients prescribed Rosuvastatin, Atorvastatin, or Pravastatin had lower HRs of AD onset than those prescribed Simvastatin. The risks of AD onset in patients prescribed Rosuvastatin, Atorvastatin, and Pravastatin were approximately 35%, 30%, and 12% lower compared to those prescribed Simvastatin, respectively. This result was consistent with an early report that Pravastatin, but not Simvastatin, was associated with a reduced risk of AD onset (50). Our results support the previous notion that individual Statins may contribute in unique ways to the central nervous system (51). The mechanisms by which the cholesterol-lowering effects of Statins contributes to the pathogenic process of AD remains unclear. It is unclear whether the possible effects of Statin use mainly works through brain cholesterol metabolism, peripheral cholesterol metabolism, or both. A clear understanding of the relationship between brain cholesterol homeostasis and AD is yet to be fully elucidated.

The comparative effectiveness against AD among Beta Blockers trails behind the development of newer Beta Blockers. In this study Carvedilol and Metoprolol were associated with reduced risk of AD compared to Propranolol.

Our study is subject to several limitations. First, the identification of AD clinical diagnoses was based on the ICD coding system, which is a widely accepted method for identifying medical conditions in large-scale administrative health data. Although comorbidities are often under-reported in administrative data, the validity of using ICD codes to study neurologic conditions is generally consistent with that of patient chart data for the recording of comorbidities (52, 53, 54). Previous analyses have indicated a high level of specificity in identifying AD and dementia cases using ICD codes across several studies (over 84%) (54). Sensitivity was found to be higher when both inpatient and outpatient records were taken into account based on ICD codes (54), which is a similar approach adopted in our study. The underestimation of Alzheimer’s disease and other dementia prevalence through ICD coding in the VA healthcare system may be attributed to the excessive use of non-specific dementia codes (55). The change of coding systems from ICD 9 to ICD 10 during the study period may have had a limited impact on the result since the validity of ICD 10 was generally similar to that of ICD 9 (56).

Second, our study population consists of a large group of Veterans who use the VA healthcare system and are largely male, exhibiting more physical and mental health conditions than would be found in a general community-wide population. In general, our cohort of non-AD subjects had a slightly higher prevalence of several comorbidity conditions at a baseline of 65 years or older than the cohort of AD patients.

Third, residual confounding factors and unmeasured variables may exist even though we have incorporated the adjustment for potential known confounders, which is common in observational studies.

Fourth, we do not know the potential mechanisms of action for the combined therapies, other than their effects on their known indications, that made some of them superior to the others. The candidate combination therapy has yet to be fully elucidated for its impact on AD progression and alteration of AD-related biomarkers.

Fifth, the combined use of medications should be carefully examined to identify the nature of any potential beneficial effects and minimize adverse drug interactions. For example, strong evidence supports the concept that a combined therapy of ACEI and ARB could significantly worsen renal failure in patients with chronic kidney disease (57). Nevertheless, concurrent use of two or more medications with different underlying mechanisms could increase the success rate of drug repositioning because of the possible synergistic effects, as well as the reduction or delay of the development of drug resistance (22).

While we do not believe our findings justify a change in clinical practice, these results warrant further investigation using additional cohorts to determine if the future findings are comparable. Future efforts to validate these combination drugs in clinical settings when new AD therapy is offered will provide the a reliable prediction about the likelihood of conversion from the pre-symptomatic stage to the clinical stage of AD for subjects who have been taking combined medication therapies.

## Supplemental Materials

Additional material Supplementary PDF file supplied by authors.

### Supporting information

**Table S1.** Overall disease prevalence for the overall sample without medication usage restriction

Comorbidities	AD (%) N=73,605	Non-AD (%) N=660,314
**Hypertension**	60523 (82.23%)	508518 (77.01%)
**Hyperlipidemia**	57727 (78.43%)	494975 (74.96%)
**Depression**	33472 (45.48%)	243461 (36.87%)
**Post-traumatic stress disorder**	8900 (12.09%)	112911 (17.1%)
**Anxiety**	17564 (23.86%)	155868 (23.61%)
**Lung disease**	24313 (33.03%)	218072 (33.03%)
**Alcohol**	7539 (10.24%)	121614 (18.42%)
**Diabetes**	27342 (37.15%)	245660 (37.2%)
**Coronary artery disease**	33619 (45.67%)	229466 (34.75%)
**Schizophrenia**	2488 (3.38%)	20410 (3.09%)
**Bipolar**	5748 (7.81%)	49225 (7.45%)
**Sleep disorder**	12157 (16.52%)	101537 (15.38%)
**Stroke**	14573 (19.8%)	68183 (10.33%)
**Peripheral arterial disease**	17712 (24.06%)	117535 (17.8%)
**Hypothyroidism**	12344 (16.77%)	79236 (12%)
**Cancer**	14312 (19.44%)	124198 (18.81%)
**Cardiac dysrhythmia**	24798 (33.69%)	156365 (23.68%)
**Congestive heart failure**	13678 (18.58%)	96665 (14.64%)
**Liver disease**	1852 (2.52%)	31851 (4.82%)
**Renal failure**	15417 (20.95%)	104860 (15.88%)
**Traumatic brain injury**	1795 (2.43%)	13655(2.06%)
**Substance use disorders**	2465 (3.35%)	64496 (9.77%)

Note: this table shows the prevalence of different diseases in the data pool where we extract our current sample in this study.
