# Supplementary material for: The Comparative Effectiveness of Monotherapy and Combination Therapies: Impact of Angiotensin Receptor Blockers on the Onset of Alzheimer’s Disease
Source: JAR Life. 2023 Jun 20;12:35–45. doi: 10.14283/jarlife.2023.8 (PMC10333644; doi:10.14283/jarlife.2023.8)

# Supporting information

**Table S1.** Overall disease prevalence for the overall sample without medication usage restriction

| Comorbidities | AD (%)  N=73,605 | Non-AD (%)  N=660,314 |
| --- | --- | --- |
| Hypertension | 60523 (82.23%) | 508518 (77.01%) |
| Hyperlipidemia | 57727 (78.43%) | 494975 (74.96%) |
| Depression | 33472 (45.48%) | 243461 (36.87%) |
| Post-traumatic stress disorder | 8900 (12.09%) | 112911 (17.1%) |
| Anxiety | 17564 (23.86%) | 155868 (23.61%) |
| Lung disease | 24313 (33.03%) | 218072 (33.03%) |
| Alcohol | 7539 (10.24%) | 121614 (18.42%) |
| Diabetes | 27342 (37.15%) | 245660 (37.2%) |
| Coronary artery disease | 33619 (45.67%) | 229466 (34.75%) |
| Schizophrenia | 2488 (3.38%) | 20410 (3.09%) |
| Bipolar | 5748 (7.81%) | 49225 (7.45%) |
| Sleep disorder | 12157 (16.52%) | 101537 (15.38%) |
| Stroke | 14573 (19.8%) | 68183 (10.33%) |
| Peripheral arterial disease | 17712 (24.06%) | 117535 (17.8%) |
| Hypothyroidism | 12344 (16.77%) | 79236 (12%) |
| Cancer | 14312 (19.44%) | 124198 (18.81%) |
| Cardiac dysrhythmia | 24798 (33.69%) | 156365 (23.68%) |
| Congestive heart failure | 13678 (18.58%) | 96665 (14.64%) |
| Liver disease | 1852 (2.52%) | 31851 (4.82%) |
| Renal failure | 15417 (20.95%) | 104860 (15.88%) |
| Traumatic brain injury | 1795 (2.43%) | 13655(2.06%) |
| Substance use disorders | 2465 (3.35%) | 64496 (9.77%) |

Note: this table shows the prevalence of different diseases in the data pool where we extract our current sample in this study.

**Figure S1.** **Variable importance ranking of medication combinations from the Random Forest model.**

The importance was measured by the mean decrease in accuracy (from the largest to the smallest). A higher mean decrease in accuracy is indicative of a more important variable, and the combinations are ordered from top to bottom as most to least important in predicting the development of AD.


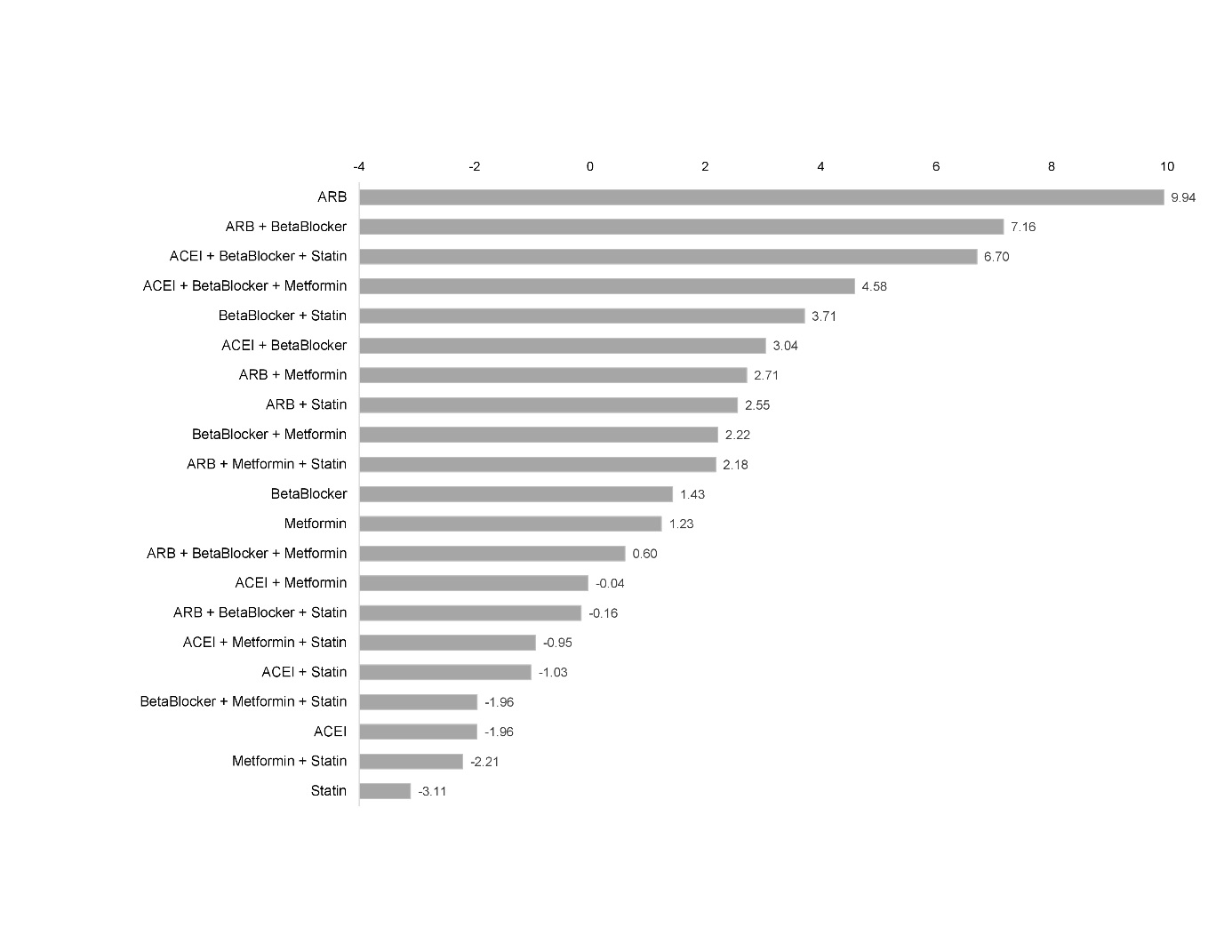


**Figure S2**: Receiver operating characteristic (ROC) curves of the random forest model that predicts the development of AD.


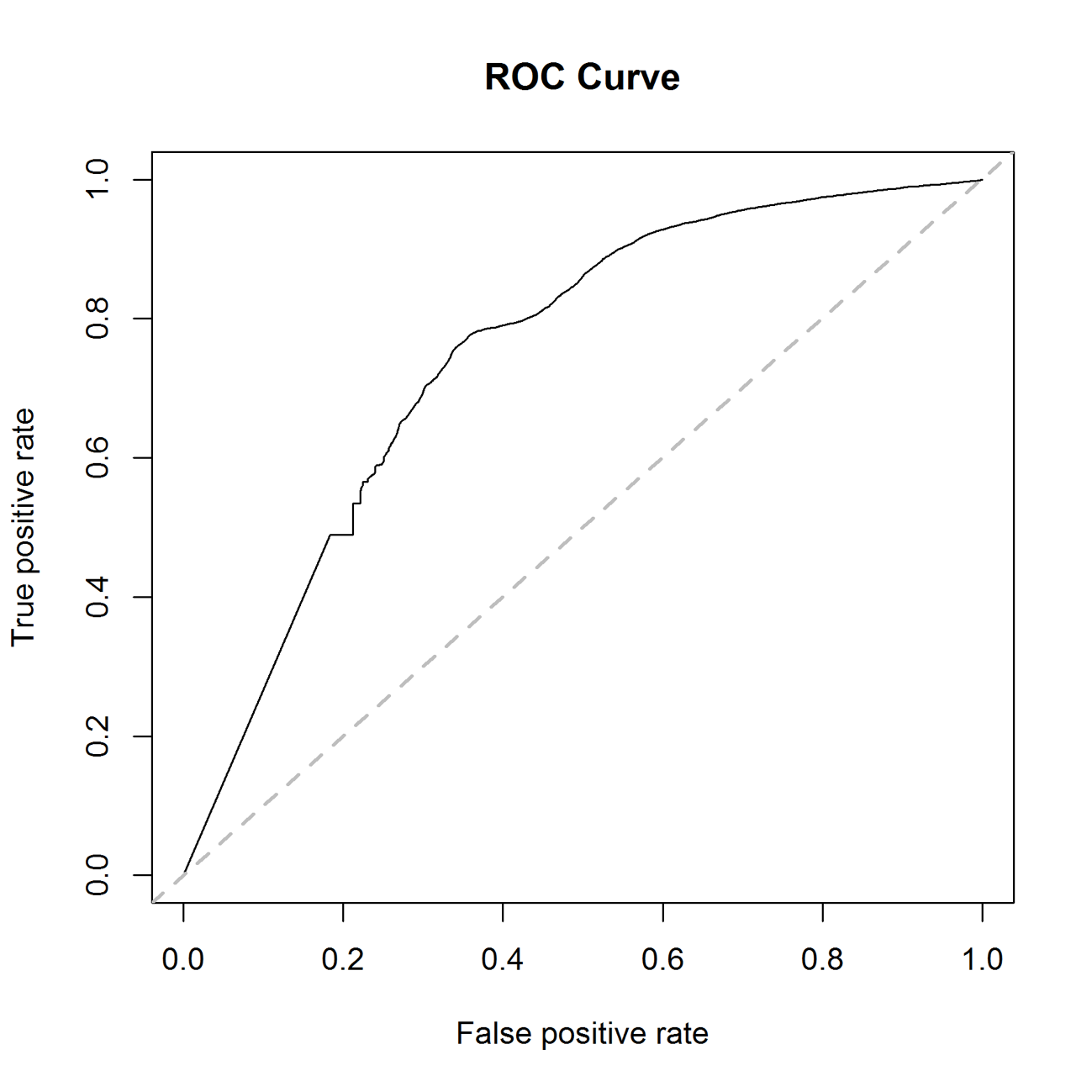

Supplement: Additional material — Supplementary PDF file supplied by authors. [file jarlife-12-008-S1.docx]
